# Supplementary material for: HIVprotI: an integrated web based platform for prediction and design of HIV proteins inhibitors
Source: J Cheminform. 2018 Mar 9;10:12. doi: 10.1186/s13321-018-0266-y (PMC5845081; doi:10.1186/s13321-018-0266-y)
Supplement: Supplementary file 2 — Additional file 2. Source code of HIVProtI web server. [file 13321_2018_266_MOESM2_ESM.zip › HIVprotI_Source-code/hivprotI/weka-3-6-6/doc/weka/gui/beans/UserRequestAcceptor.html]

UserRequestAcceptor


---


|  |  |  |
| --- | --- | --- |
| **Overview** \| **Package** \| **Class** \| **Tree** \| **Deprecated** \| **Index** \| **Help** \| **Weka's home** | |  |
| **PREV CLASS**   **NEXT CLASS** | **FRAMES**    **NO FRAMES**     **All Classes** |  |
| SUMMARY: NESTED \| FIELD \| CONSTR \| METHOD | DETAIL: FIELD \| CONSTR \| METHOD |  |


---


## weka.gui.beans Interface UserRequestAcceptor

**All Known Implementing Classes:**: Associator, AttributeSummarizer, Classifier, ClassifierPerformanceEvaluator, Clusterer, ClustererPerformanceEvaluator, CostBenefitAnalysis, CrossValidationFoldMaker, DataVisualizer, Filter, GraphViewer, MetaBean, ModelPerformanceChart, ScatterPlotMatrix, StripChart, TextViewer, TrainTestSplitMaker

---

``` public interface UserRequestAcceptor ```

Interface to something that can accept requests from a user to perform
some action

**Since:**
:   1.0

**Version:**
:   $Revision: 1.3 $

**Author:**
:   Mark Hall

---

| **Method Summary** | |
| --- | --- |
| `java.util.Enumeration` | `enumerateRequests()`             Get a list of performable requests |
| `void` | `performRequest(java.lang.String requestName)`             Perform the named request |

**Method Detail**

### enumerateRequests

```
java.util.Enumeration enumerateRequests()
```

:   Get a list of performable requests

    :   **Returns:**: an `Enumeration` value

---


### performRequest

```
void performRequest(java.lang.String requestName)
```

:   Perform the named request

    :   **Parameters:**: `requestName` - a `String` value **Throws:**: `java.lang.IllegalArgumentException` - if an error occurs


---


|  |  |  |
| --- | --- | --- |
| **Overview** \| **Package** \| **Class** \| **Tree** \| **Deprecated** \| **Index** \| **Help** \| **Weka's home** | |  |
| **PREV CLASS**   **NEXT CLASS** | **FRAMES**    **NO FRAMES**     **All Classes** |  |
| SUMMARY: NESTED \| FIELD \| CONSTR \| METHOD | DETAIL: FIELD \| CONSTR \| METHOD |  |


---
